# Soluble Endoglin Level Increase Occurs Prior to Development of Subclinical Structural Vascular Alterations in Diabetic Adolescents

**DOI:** 10.4274/jcrpe.2906

**Published:** 2016-09-01

**Authors:** Hamdi Cihan Emeksiz, Aysun Bideci, Çağrı Damar, Betül Derinkuyu, Nurullah Çelik, Esra Döğer, Özge Yüce, Mehmet Cüneyt Özmen, Mahmut Orhun Çamurdan, Peyami Cinaz

**Affiliations:** 1 Gazi University Faculty of Medicine, Department of Pediatric Endocrinology, Ankara, Turkey; 2 Gazi University Faculty of Medicine, Department of Radiology, Ankara, Turkey; 3 Gazi University Faculty of Medicine, Department of Ophthalmology, Ankara, Turkey

**Keywords:** type 1 diabetes mellitus, subclinical atherosclerosis, soluble endoglin, adolescents

## Abstract

**Objective:**

Soluble endoglin (S-endoglin) has been implicated as a potential marker of endothelial dysfunction (ED) and was reported to be elevated in diabetic adults, correlating with the severity of diabetic vasculopathy. However, circulating S-endoglin and its association with other markers of ED have not been formerly analyzed in the first decade of diabetes onset in adolescents with type 1 diabetes mellitus (T1DM).

**Methods:**

Fifty-eight adolescents with moderately/poorly controlled T1DM were included in this study and twenty-nine healthy adolescents served as controls. The diabetic group was divided into two groups based on the presence of microalbuminuria, as the microalbuminuria group (n=15) and the normoalbuminuria group (n=43). Functional vascular alterations were evaluated by measuring serum S-endoglin and plasma nitric oxide (NO) concentrations, the flow-mediated dilatation (FMD) of the brachial artery. Carotid intima media thickness (CIMT) was measured for evaluation of structural vascular alterations.

**Results:**

The S-endoglin and NO levels of both microalbuminuria and normoalbuminuria groups were higher than those of the control group (for S-endoglin, p=0.047 and p<0.001; for NO, p=0.004 and p=0.006, respectively). The FMD percent was lower in the microalbuminuria group compared to the normoalbuminuria and control groups (p=0.036 and p=0.020, respectively). There were negative correlations between S-endoglin concentration and FMD percent (r=-0.213, p=0.051) and between serum S-endoglin concentration and albumin excretion rate (r=-0.361, p=0.005). No significant differences were found in CIMT among any of the groups (p=0.443).

**Conclusion:**

In adolescents with T1DM, S-endoglin concentrations might increase in parallel to the deterioration in endothelial function before subclinical structural vascular alterations become evident.

**WHAT IS ALREADY KNOWN ON THIS TOPIC?**

Soluble endoglin molecule has been suggested as a potential biomarker of endothelial dysfunction since its concentration was found elevated in several diseases with vascular involvement. Recently, concentration of this molecule has been found to be increased in diabetic adults, correlating with the severity of diabetic vascular insult.

**WHAT THIS STUDY ADDS?**

We analyzed soluble endoglin concentration and its relation with other suggested markers of endothelial dysfunction in the first decade of diabetes onset in adolescents with type 1 diabetes mellitus.

## INTRODUCTION

Hyperglycemia causes subclinical functional and structural vascular alterations associated with premature atherosclerosis even in childhood type 1 diabetes mellitus (T1DM). It precipitates the early emergence of endothelial dysfunction (ED) as a subclinical functional vascular alteration representing the initial step toward the atherosclerotic process that promotes the development of cardiovascular diseases (CVDs) and also of microvascular complications in patients with T1DM. Several biomarkers and also radiological methods including measurement of flow-mediated dilatation (FMD) of the brachial artery and carotid intima media thickness (CIMT) have been used to assess diabetes-related early vascular alterations. Changes in the amount and/or bioavailability of nitric oxide (NO) molecule, the major agent that contributes to the anti atherosclerotic effects of endothelium, constitute one of the early findings of ED (1,2,3,4).

Another molecule which has been implicated in the regulation of endothelial function is endoglin, a 180 Kda homodimeric integral membrane glycoprotein serving as a receptor for the transforming growth factor-β (TGF-β) superfamily (5,6,7,8). Demonstration of increased endoglin expression in atherosclerotic plaques suggested the participation of endoglin in the atherosclerotic process (8). Furthermore, a soluble form of endoglin (S-endoglin), generated by the cleavage of the extracellular domain of the entire endoglin molecule, has also been suggested as a marker of ED and reported to be increased in the serum of patients with preeclampsia, hypercholesterolemia, and atherosclerosis (9,10,11,12). In addition, the circulating level of S-endoglin has also been shown to be elevated in patients with type 2 diabetes mellitus and considered as an indicator of diabetes-related vascular pathologies (13). However, it is not known whether circulating S-endoglin level changes in the first decade of T1DM onset is a potential early marker of ED in adolescents with T1DM.

A widely used and reliable radiological method to measure endothelial function in patients with T1DM is FMD of the brachial artery (14,15). Numerous studies have consistently reported that children with T1DM have decreased FMD percentage relative to healthy controls (15,16). Furthermore, some studies reported increased CIMT, as a next stage, representing the occurrence of subclinical structural vascular alteration in young subjects with T1DM (16,17).

Circulating S-endoglin level was found to be elevated in diabetic adults correlating with the severity of diabetic vascular changes that suggested S-endoglin as a potential marker of ED. However, circulating S-endoglin level and its relation with other suggested markers of ED have not been investigated in the first decade of T1DM onset in diabetic adolescents. Therefore, in the present study, we evaluated subclinical vascular alterations radiologically by ultrasonographic measurement of FMD and CIMT, and biochemically by measurement of plasma NO level along with serum S-endoglin level in adolescents with T1DM.

## METHODS

This cross-sectional study was performed during the period September 2013 to February 2014 and included 58 adolescents with T1DM followed in Gazi University Faculty of Medicine Hospital, Pediatric Endocrinology Clinic and 29 group-matched healthy controls. Data on age, gender, duration of diabetes, insulin regimen, and daily requirement for insulin and mean annual glycated hemoglobin (HbA_1C_) levels were collected from the medical records. Mean HbA_1C_ levels of the follow-up period (f-HbA_1C_) and the preceding year (py-HbA_1C_) were calculated. HbA_1C_ levels at the time of T1DM diagnosis were excluded while calculating f-HbA_1C_ levels. Diabetic adolescents (n=58) were divided into two groups based on the presence of persistent microalbuminuria, as the microalbuminuria group (n=15) and the normoalbuminuria group (n=43). Persistent microalbuminuria was defined as a urinary albumin excretion rate (AER) between 30-300 mg/dL in at least two of three 24-hour urine samples over a 3-month period (18). None of the diabetic adolescents were receiving any treatment with other drugs except insulin.

All participants were subjected to physical examination. Height was measured to the nearest centimeter using a Harpenden stadiometer (Holtain Instruments Ltd, UK). Weight was measured unclothed to the nearest 0.1 kg using a calibrated balance scale. Body mass index (BMI) was calculated using the weight (kg)/height (m^2^) equation. Standard deviation scores (SDS) for weight, height, and BMI were calculated using the reference values for Turkish children (19). Measurements of blood pressure were performed in all cases after a period of resting and were repeated 3 times with 10-minute intervals. Subjects with systolic and/or diastolic blood pressure above the 95th percentile were accepted as hypertensive. Pubertal status of each case was defined according to Tanner criteria. All subjects were nonsmokers and were normotensive, had normal plasma lipids, liver and renal functions, plasma electrolyte levels, and a normal blood count. Exclusion criteria of the patients with T1DM included smoking, dyslipidemia, hypertension, and presence of a chronic disease other than T1DM. The healthy control adolescents included in this study were volunteers in Gazi University Faculty of Medicine Hospital. Their inclusion criteria were good health, no known history of chronic disease, and no medications which might influence cardiovascular function, glucose, or lipid metabolism. The study protocol was approved by Gazi University Faculty of Medicine Clinical Trial Ethics Committee. Informed consent and assent were obtained from all subjects and their parents.

Peripheral venous blood samples were obtained to determine fasting plasma glucose (FPG), lipids [total cholesterol, low density lipoprotein (LDL), high density lipoprotein (HDL), triglycerides], blood urea nitrogen (BUN), creatinine, aspartate aminotransferase (AST), alanine aminotransferase (ALT), NO, and serum S-endoglin concentrations after an overnight 12-hour fast. AER was measured in 24-hour urine samples. These parameters were evaluated on a blind basis in Gazi University Faculty of Medicine Hospital Biochemistry Laboratory using standard automatized techniques. Plasma glucose concentrations were measured by glucose oxidase reaction. BUN, creatinine, AST, ALT, and lipid concentrations were measured by spectrophotometric methods, and albumin concentrations in 24-hour urine samples were measured by immunoturbidimetry on an autoanalyzer (Beckman Coulter, La Brea, CA, USA).

Subclinical functional vascular alterations were evaluated by measuring plasma NO and serum S-endoglin concentrations and FMD of the brachial artery, whereas subclinical structural vascular alteration was evaluated by measuring CIMT. Serum S-endoglin concentrations were measured by using an enzyme-linked immunosorbent assay method (Human Endoglin; R&D Systems, Minneapolis, MN, USA). Plasma NO concentrations were determined using the Griess reaction by measuring combined oxidation products of NO, plasma nitrite (NO2), and nitrate (NO3) after reduction with nitrate reductase in a colorimetric assay (Cayman Inc., Ann Arbor, Michigan, USA). Before initiation of the assay to prepare sample solutions, hemoglobin and proteins were removed using a membrane filter (Amicon Ultra 10 Kda Ultracel; Millipore, Darmstadt, Germany). The intra- and inter-assay coefficients of variation for S-endoglin were 3% and 6.3% and for NO, 2.6% and 4.2%, respectively.

### Flow-Mediated Dilatation Measurements

Patients were instructed to avoid any food or drink containing caffeine before the procedures as such substances may interfere with endothelial functions. After the patients had rested for ten minutes in supine position, the brachial artery was located in the antecubital fossa and its basal diameter was measured with a 12 MHz high-resolution linear probe. Following the optimal basal measurement, systolic blood pressure was increased to 250 mmHg with the instrument of the cuff placed above the measurement site, and the ischemia and shear stress were sustained for 5 minutes. The cuff was then deflated and sequential re-measurements of the brachial artery diameter were done with 30-second intervals for 2 minutes. The peak brachial artery diameter that was measured after the deflation of the cuff was recorded. Finally, the percentage flow-mediated dilatation index (FMD%) was calculated by dividing the maximum arterial diameter change by the basal arterial diameter (14,20).

### Carotid Intima-Media Thickness Measurements

After a period of 10-minute rest, CIMT was measured using the 12 MHz linear probe while the patients were in supine position with slight extension of the head towards the opposite of the carotid artery of interest. An optimal longitudinal, 2-dimensional image of the distal common carotid artery was frozen on screen. The CIMT was determined by taking the mean of all three measurements (21).

### Statistical Analysis

After entering the data using the Statistical Package for the Social Sciences (SPSS) Version 18.0 software (SPSS Inc., Chicago, IL, USA), the analysis of the results were performed using percentage distribution for qualitative data and median interquartile range (IQR) or mean (standard deviation) for quantitative data. The statistical tests used were the Shapiro-Wilks test for normality, the chi-square test for qualitative data comparison of groups, and the independent samples test, Mann-Whitney U-test, one way analysis of variance (ANOVA), and Kruskal-Wallis test for quantitative data comparison of groups, as appropriate. The correlation between quantitative data was calculated by Spearman rank correlation. A p-value less than 0.05 was considered statistically significant.

## RESULTS

Eighty-seven adolescents (40 female) were included in the study. Except for FPG concentrations, there were no significant differences for demographic and metabolic characteristics among the groups (p>0.05 for all, except FPG). No statistically significant difference was found for systolic and diastolic pressures between the groups (p=0.483 and p=0.625, respectively). None of the participants had hypertension. Neither mean f-HbA_1C_ nor mean py-HbA_1C_ levels significantly differed between the microalbuminuria and normoalbuminuria groups (Table 1).

**Figure 1:**
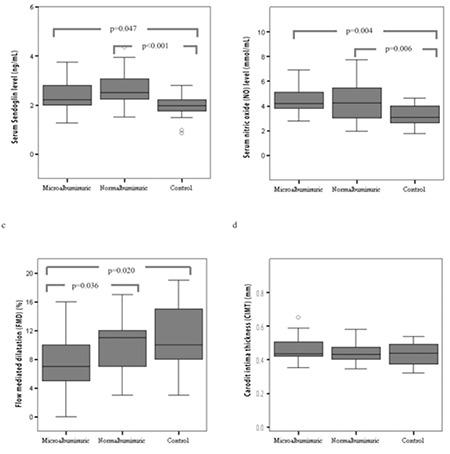
Comparison of NO, S-endoglin, FMD, and CIMT measurements among groups. Kruskal-Wallis test over all groups for NO p<0.05 and for CIMT p>0.05. One way ANOVA test over all groups for S-endoglin and FMD p<0.05. p-values of pairwise comparisons (Mann-Whitney U test and independent samples t-test) are shown in the diagram. NO: nitric oxide, S-endoglin: soluble endoglin, FMD: flow-mediated dilatation, CIMT: carotid intima-media thickness, ANOVA: analysis of variance

Serum S-endoglin concentration significantly differed among the groups (p<0.001). Both normoalbuminuria and microalbuminuria groups had significantly higher S-endoglin concentrations [2.50 (2.19-3.09) ng/mL and 2.21 (1.91-2.90) ng/mL, respectively] compared to the control group [1.97 (1.72-2.23) ng/mL] (p<0.001 and p=0.047, respectively) (Figure 1a). S-endoglin concentration was higher in the normoalbuminuria group compared to the microalbuminuria group but did not reach statistical significance (p=0.108).

The difference in plasma NO concentration was significant among the groups (p=0.005). NO concentrations of both microalbuminuria and normoalbuminuria groups [41.8 (37.7-51.2) µmol/L and 42.5 (30.1-54.9) µmol/L, respectively] were significantly higher compared to the control group [30.8 (25.7-40.4) µmol/L] (p=0.004 and p=0.006, respectively) (Figure 1b). No significant difference was detected in NO level between the microalbuminuria and normoalbuminuria groups (p=0.703).

FMD% significantly differed among the groups (p=0.032). The microalbuminuria group had a significantly lower FMD% (7.53±3.29%) compared to the control and normoalbuminuria groups (10.9±4.01% and 9.93±3.51%, respectively) (p=0.020 and p=0.036, respectively) (Figure 1c). The FMD% was lower in the normoalbuminuria group than in the control group, but the difference was not statistically significant (p=0.306). The CIMT was slightly higher in the microalbuminuria group [0.44 (0.42-0.55) mm] compared to the normoalbuminuria group [0.43 (0.40-0.48) mm] and the control group [0.43 (0.37-0.48) mm], but this difference did not reach statistical significance (p=0.443) (Figure 1d). There were no gender differences in S-endoglin, NO, FMD, and CIMT measurements in the diabetic group or in the overall study population (p>0.05 for all).

**Figure 2:**
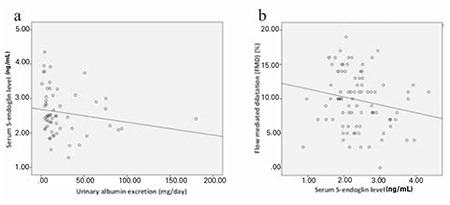
Relationship of serum S-endoglin level with urinary albumin excretion (Figure 2a; r=-0.361) and flow-mediated dilatation % (Figure 2b; r=-0.213). S-endoglin: soluble endoglin

Significant negative correlations were found between serum S-endoglin concentration and AER (r=-0.361, p=0.005) as well as between S-endoglin concentration and FMD% (r=-0.213, p=0.051) (Figure 2). There was a weak positive association between S-endoglin and NO concentrations (r=0.203, p=0.059). Except for these, there were no associations between the parameters evaluated in this study (p>0.05 for all).

## DISCUSSION

In the first decade of T1DM onset, we found significantly increased plasma NO concentrations as well as increased S-endoglin concentrations in both microalbuminuria and normoalbuminuria groups relative to the control group. On the other hand, there were no significant differences in CIMT among any of the groups. Furthermore, we detected a weak negative association between S-endoglin concentration and FMD% and a weak positive association between S-endoglin and NO concentrations. In the light of these data, we suggested that in adolescents with T1DM, S-endoglin concentrations might increase in parallel to the deterioration in endothelial function before subclinical structural vascular alterations became evident. Moreover, considering the inverse association between circulating S-endoglin and AER and the presence of slightly lower S-endoglin concentration in the microalbuminuria group compared to the normoalbuminuria group, we speculate that exposure to T1DM initially may give rise to an increase in serum S-endoglin concentrations; however, with the development of microalbuminuria, a relative decline in circulating S-endoglin concentrations might be observed. All these findings suggest that increased circulating S-endoglin may be an early indicator of diabetes-related functional vascular alterations and even might precede the development of microalbuminuria, before the manifestation of subclinical structural vascular alterations in adolescents with T1DM.

Changes in the bioavailability of NO molecule have been reported as an indicator of ED in diabetic vasculopathy (22). However, while increased levels of NO production were detected in early diabetes, as the duration of diabetes increases, reduced blood NO concentrations were measured in diabetic patients (3,4,23,24,25). Accordingly, we found significantly higher plasma NO concentrations in both microalbuminuria and normoalbuminuria groups relative to the control group in the first decade of T1DM onset. Although the reasons are not fully known, induction of inducible NO synthase synthesis and upregulation of NO production as a response to its reduced bioavailability due to enhanced free radical formation are suggested mechanisms for increased NO production in early diabetes (26,27).

In a considerable number of studies, it has been reported that impaired FMD, as a reliable indicator of ED, may become evident even a few years after the onset of T1DM (15,16). We found significantly reduced FMD in the microalbuminuria group compared to both normoalbuminuria and control groups, whereas such a significant difference was not detected between the normoalbuminuria and control groups. It was widely assumed that FMD was a direct indicator of NO bioavailability in the endothelium. However, considerable evidence showed that FMD response in the conduit arteries is not merely NO-dependent (28,29). Although controversy exists, certain well-designed studies reported that substances used to block NO synthesis were not able to deteriorate the FMD response (30). Moreover, it has been demonstrated that endothelial NO synthase knockout mice were still capable of dilating their arteries as a response to shear stress due to release of additional vasodilatory molecules, such as prostacyclin (PGI2) and endothelial-derived hyperpolarizing factor which may contribute to FMD (31). On the other hand, impact of these molecules and NO on FMD may vary according to shear stress creation technique, vascular bed, and diseased states (28,29). In line with these data, in our study, despite the presence of more impaired FMD in the microalbuminuria group relative to the normoalbuminuria group, plasma NO concentrations did not significantly differ between the microalbuminuria and normoalbuminuria groups, and furthermore, we did not find a correlation between plasma NO concentration and FMD%. According to the above-mentioned findings, it may be suggested that circulating NO level alone, as an indicator of ED, may not be sufficient to predict the severity of ED in adolescents with T1DM.

Recently, S-endoglin has been implicated as a potential marker of ED (6). Hypoxia and oxidative stress are considered triggers of S-endoglin release, which, in turn, inhibits the anti-atherogenic effects induced by TGF-β (32,33). Although a more recent study has demonstrated that increased S-endoglin level per se is not capable of inducing ED in an animal model, the authors indicated that their finding does not rule out the possibility that S-endoglin might contribute to alteration of endothelial function in the presence of other risk factors related to CVDs (34). Increased circulating concentrations of S-endoglin were reported to be associated with vascular damage in several disease states including preeclampsia, hypercholesterolemia, and atherosclerosis (10,11,12). A similar relationship between increased serum S-endoglin concentrations and diabetes-related vascular disorders and a positive association between circulating S-endoglin and ED have also been demonstrated in an adult study from Spain (13). In addition, glucagon-like peptide-1 has been shown to reduce plasma S-endoglin levels and oxidative stress in patients with T1DM, presumably due to its intracellular antioxidant activity (35). Our study is the first to assess serum S-endoglin concentrations as a potential early marker of ED representing subclinical vascular alterations in a young cohort with T1DM. It is known that children with T1DM may develop ED within the first decade after its onset and that diabetes-related structural vascular alterations occur after the development of diabetes-related functional vascular alterations. In our study, there was no significant difference in CIMT among the three groups, however, as was also true for NO concentrations, significantly increased circulating S-endoglin concentrations were measured in both microalbuminuria and normoalbuminuria groups relative to the control group. Moreover, we detected a weak linear correlation between S-endoglin and NO concentrations as well as a weak inverse correlation between S-endoglin and FMD%; these are findings which might favor the probable association of S-endoglin with ED in T1DM. In the light of these data, we suggest that in adolescents with T1DM, S-endoglin concentrations might increase in parallel to the deterioration in endothelial function prior to the appearance of subclinical structural vascular alterations. However, long-term prospective studies investigating the association of S-endoglin with other indicators of endothelial function are needed to confirm these findings.

Li et al (36) found S-endoglin and TGF-β1 concentrations of patients with severe coronary atherosclerosis significantly lower than those of patients with mild coronary atherosclerosis and those of healthy controls. They proposed that in the early stages of atherosclerosis, circulating concentration of S-endoglin increases due to damage of endothelial cells, but with the progression of the atherosclerotic process, S-endoglin concentration decreases due to elevated levels of S-endoglin/TGF-β1 complexes in blood serum (36). Likewise, with the progression of ED during the course of T1DM, alterations in serum S-endoglin concentration may be observed. S-endoglin may be important in only certain stages of diabetes-related vascular insult. The circulating concentration of S-endoglin may decrease over time and this decrease may be due to decreased production or enhanced complex formation with other yet unidentified substances in the circulation. In our study, the highest S-endoglin concentration was found in the normoalbuminuria group. As compared to the normoalbuminuria group, the microalbuminuria group had an insignificantly lower S-endoglin concentration. However, this latter group had significantly reduced FMD which confirms the presence of more evident ED in the microalbuminuria group. As known, microalbuminuria is an indicator of generalized ED and is regarded as a common pathway of injury to both renal and systemic vascular beds (37). Of note, we detected a significant inverse association between AER and serum S-endoglin concentration. According to the above-mentioned results, it may be speculated that in adolescents with T1DM, exposure to a diabetic milieu might initially lead to an increase in circulating concentrations of S-endoglin, but, with the development of microalbuminuria, a relative decrease may be observed in these levels.

Our study has several limitations. First, the small sample size of the cohort might have undermined the power of this study while making conclusions due to the alterations in markers used in the evaluation of subclinical atherosclerosis. Absence of certain associations could also be related to this limitation. Second, our study design was a cross-sectional one which may not provide definite information about cause-and-effect relationships. Therefore, the associations of S-endoglin with ED and the long-term micro- and macrovascular complications of T1DM need to be analyzed prospectively.

Our findings suggest that within the first decade of T1DM onset, circulating concentrations of S-endoglin may increase before an increase in CIMT and with the rise of AER above a definite critical level which might be closer to the lower range of microalbuminuria; a comparative decrease in the circulating S-endoglin level may be observed in adolescents with T1DM. However, long-term prospective studies measuring S-endoglin concentrations with recognized indicators of ED are needed to better elucidate the relationship of S-endoglin with atherosclerotic process and microvascular complications of T1DM.

## Ethics

Ethics Committee Approval: This study was approved by the Scientific Review Committee of Gazi University Faculty of Medicine (approval number: 25901600/5447), Informed Consent: It was taken.

Peer-review: Externally peer-reviewed.

## Figures and Tables

**Table 1:**
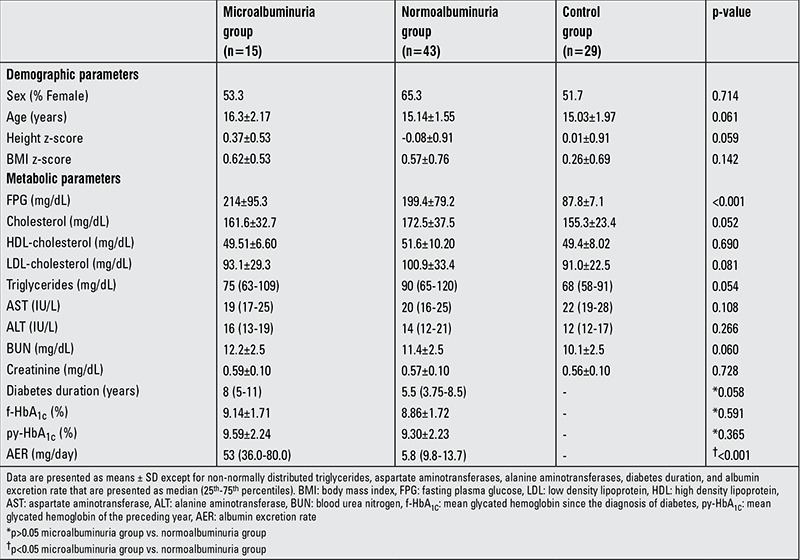
Demographic and metabolic characteristics of the study subjects
